# Menschen mit Migrationsgeschichte in der COVID-19-Pandemie: Zusammenhänge zwischen der Lebenszufriedenheit und indirekten sozioökonomischen Pandemiefolgen

**DOI:** 10.1007/s00103-023-03741-0

**Published:** 2023-07-20

**Authors:** Carmen Koschollek, Susanne Bartig, Stephan Müters, Laura Goßner, Marleen Bug, Lena Goerigk, Claudia Hövener, Katja Kajikhina

**Affiliations:** 1 Abteilung für Epidemiologie und Gesundheitsmonitoring, Robert Koch-Institut, Berlin, Deutschland; 2 Institut für Arbeitsmarkt- und Berufsforschung, Nürnberg, Deutschland; 3 Abteilung für Infektionsepidemiologie, Robert Koch-Institut, Berlin, Deutschland; 4 Abteilung für Epidemiologie und Gesundheitsmonitoring, Fachgebiet 28 Soziale Determinanten der Gesundheit, Robert Koch-Institut, General-Pape-Str. 62–66, 12101 Berlin, Deutschland

**Keywords:** Migration, Soziale Ungleichheit, SARS-CoV-2, Gesundheit, Arbeitsbedingungen, Migration, Social inequality, SARS-CoV-2, Health, Working conditions

## Abstract

**Einleitung:**

Nicht nur Risiken für eine SARS-CoV-2-Infektion und schwere bis tödliche Verläufe sind sozial ungleich verteilt, sondern auch Arbeitsplatz- und Einkommensverluste infolge der Eindämmungsmaßnahmen. Für Menschen mit Migrationsgeschichte zeigen sich ebenfalls erhöhte Risiken, von solchen indirekten sozioökonomischen Pandemiefolgen betroffen zu sein. Ziel dieses Beitrages ist es, Zusammenhänge zwischen indirekten sozioökonomischen Pandemiefolgen und der Lebenszufriedenheit von Menschen mit ausgewählten Staatsangehörigkeiten zu untersuchen.

**Methoden:**

Analysiert wurden Daten der multimodalen, mehrsprachigen Befragungsstudie Gesundheit in Deutschland aktuell: Fokus (GEDA Fokus), die von 11/2021 bis 05/2022 unter Menschen mit italienischer, kroatischer, polnischer, syrischer oder türkischer Staatsangehörigkeit deutschlandweit durchgeführt wurde. In multivariablen Poisson-Regressionen werden Zusammenhänge zwischen Geschlecht, Alter, Bildung, Einkommen, Deutschkenntnissen sowie Arbeitsplatz- und Einkommensverlusten und der Lebenszufriedenheit untersucht.

**Ergebnisse:**

Von 4114 Teilnehmenden berichten 64,4 % eine hohe Lebenszufriedenheit. Während ein hohes Einkommen positiv mit einer hohen Lebenszufriedenheit assoziiert ist, zeigen sich negative Assoziationen bei selbst als schlecht eingeschätzten Deutschkenntnissen sowie bei mit hoher Wahrscheinlichkeit erwarteten bzw. bereits eingetretenen Arbeitsplatz- und Einkommensverlusten.

**Diskussion:**

Der Beitrag zeigt, dass die Lebenszufriedenheit, die für eine Reihe gesundheitlicher Outcomes relevant ist, bei denjenigen geringer ist, die von Arbeitsplatz- und Einkommensverlusten betroffen sind. Es gilt, strukturelle Ursachen sozioökonomischer Benachteiligung abzubauen, um gesundheitliche Ungleichheiten zu adressieren und für künftige Krisen besser gewappnet zu sein.

**Zusatzmaterial online:**

Zusätzliche Informationen sind in der Online-Version dieses Artikels (10.1007/s00103-023-03741-0) enthalten.

## Abstract (en)

**Introduction:**

It is not only the risks of SARS-CoV‑2 infection and severe to fatal courses of the disease that are socially unequally distributed, but also job and income losses as a result of the containment measures. People with a history of migration are at increased risk of being affected by such indirect socio-economic effects of the pandemic as well. The aim of this article is to investigate the associations between indirect socio-economic effects of the pandemic and life satisfaction among people with selected citizenships.

**Methods:**

We analysed data from the multilingual and multimodal interview survey German Health Update Fokus (Gesundheit in Deutschland aktuell: Fokus; GEDA Fokus), which was conducted from November 2021 to May 2022 among people all over Germany with Croatian, Italian, Polish, Syrian or Turkish citizenship. Using multivariable Poisson regression, we analysed associations between sex, age, education, income, German language proficiency and job as well as income losses and life satisfaction.

**Results:**

Of the 4114 participants, 64.4% reported a high life satisfaction. While a higher income showed positive associations with life satisfaction, negative associations were found for lower self-assessed German language proficiency and for job and income losses that are anticipated or have already occurred.

**Discussion:**

This article shows that life satisfaction, which is relevant for multiple health outcomes, is lower among those that are affected by job and income losses. Structural causes of socio-economic disadvantages need to be reduced to address health inequalities and to be better prepared for future crises.

## Hintergrund

„Vor dem Virus sind alle gleich“, lautete eine der frühen Einschätzungen des Infektionsgeschehens mit dem neuartigen Coronavirus SARS-CoV‑2 in der Fach- und medialen Öffentlichkeit [1]. Internationale Studien zeigten hingegen alsbald, dass vor allem Personen in sozioökonomisch benachteiligten Regionen von höheren Inzidenzen und höherer Mortalität betroffen waren [2]. Für Deutschland wurden diese Befunde bereits ab der zweiten Pandemiewelle sowie im weiteren Verlauf deutlich, sowohl in ökologischen Studien als auch auf Individualebene [3–5]. Auch Menschen mit Migrationsgeschichte^1^ [6] waren häufiger von erhöhten Inzidenz- und Mortalitätsraten durch COVID-19 betroffen, wie die internationale Forschung [7–12] und erste Befunde aus Deutschland zeigten [12–14]. Zurückführen lässt sich dies nicht auf die Migrationsgeschichte selbst, sondern insbesondere auf die Lebens- und Arbeitsbedingungen [12].

Über die direkten Folgen der Pandemie – wie etwa Infektionen oder die durch COVID-19 verursachte Mortalität – hinaus, zeigen sich indirekte Folgen des Pandemiegeschehens, wie Arbeitsplatz- oder Einkommensverluste. Schwierige wirtschaftliche Entwicklungen und behördlich angeordnete Maßnahmen zur Eindämmung des Virus sorgten landesweit für Arbeitsausfälle. Auch hier stellt sich die Frage, ob diese indirekten Pandemiefolgen in verschiedenen Bevölkerungsgruppen unterschiedlich stark wirken. Im Frühsommer 2020 waren laut Erwerbstätigenbefragung des Instituts für Arbeitsmarkt- und Berufsforschung (IAB) deshalb etwa 20 % der sozialversicherungspflichtig Beschäftigten in Kurzarbeit, insbesondere Beschäftigte mit einem geringeren Haushaltseinkommen oder niedrigerer Bildung [15]. Beschäftigte im Niedriglohnsektor waren häufiger in Kurzarbeit [16], aber auch von Arbeitslosigkeit bedroht [17].

Menschen mit Migrationsgeschichte waren in Deutschland schon vor der Pandemie häufiger von Bildungsbenachteiligung, niedrigen Löhnen und von Armut oder Armutsrisiko betroffen [18]. Daten des IAB zeigen darüber hinaus, dass Menschen mit *Migrationshintergrund*, und insbesondere Geflüchtete, häufiger in befristeten Beschäftigungsverhältnissen oder Zeitarbeit tätig waren als Personen ohne *Migrationshintergrund* [19]. Außerdem arbeiten sie häufiger im Gesundheits- und Pflegebereich mit direktem Kontakt zu Patient:innen sowie in weiteren systemrelevanten Berufen [20] mit verminderten Schutzmöglichkeiten sowie auch in volatilen, krisenanfälligen Berufen [21].

Schon in der frühen Phase der Pandemie zeigten verschiedene Erhebungen, dass das Vorhandensein eines *Migrationshintergrundes* mit vermehrten Einkommenseinbußen assoziiert war [22, 23] und hier entsprechend häufiger von Sorgen über die eigene wirtschaftliche Situation berichtet wurde [23]. Wie sich diese indirekten sozioökonomischen Folgen der Pandemie auf die Gesundheit von Menschen mit Migrationsgeschichte in Deutschland auswirken, wurde bisher nicht untersucht.

Gleichwohl sind Zusammenhänge zwischen sozioökonomischer Benachteiligung sowie ungünstigen Arbeitsbedingungen und deren Auswirkungen auf die Gesundheit in der Literatur hinreichend beschrieben [24, 25]. Es kann daher angenommen werden, dass sich auch die sozioökonomischen Benachteiligungen während der Pandemie auf den Gesundheitszustand auswirken können. Die Lebenszufriedenheit ist hierfür ein guter Indikator, da sie ein „Bilanzmaß des subjektiven Wohlbefindens“ [26] darstellt. Sie spiegelt die subjektive Bewertung der eigenen aktuellen Lebenssituation wider und gilt als wichtiger Aspekt des psychischen Wohlbefindens [26–29]. Die Studienlage deutet auf Zusammenhänge zwischen geringeren Risiken für chronische Erkrankungen und geringeren Mortalitätsraten bei einer höheren Lebenszufriedenheit hin [27, 29]. In einem Review konnten mehrheitlich Zusammenhänge zwischen einer hohen Lebenszufriedenheit und einem selteneren Auftreten psychischer Störungen bei Menschen mit Migrationsgeschichte identifiziert werden [30].

Vor diesem Hintergrund ist es das Ziel dieses Beitrags, zu untersuchen, wie sich antizipierte oder bereits eingetretene Veränderungen in der Arbeits- und Einkommenssituation infolge der Maßnahmen zur Eindämmung der COVID-19-Pandemie auf die Lebenszufriedenheit von Menschen mit ausgewählten Staatsangehörigkeiten auswirken.

## Methoden

### Datenbasis: Befragungsstudie GEDA Fokus

Die vorliegenden Analysen basieren auf Daten der mehrsprachigen Befragungsstudie „Gesundheit in Deutschland aktuell: Fokus“ (GEDA Fokus; November 2021 – Mai 2022), die unter Menschen mit italienischer, kroatischer, polnischer, syrischer oder türkischer Staatsangehörigkeit im Alter von 18 bis 79 Jahren in 99 Städten und Gemeinden in ganz Deutschland am Robert Koch-Institut (RKI) durchgeführt wurde. Die Auswahl der Staatsangehörigkeitsgruppen (Grundgesamtheit) erfolgte anhand von Modellrechnungen unter Berücksichtigung der Gruppengröße sowie der Dynamik in Form von Zu- und Fortzügen [31].

Mittels einer Einwohnermeldeamtsstichprobe wurden Studienpersonen nach dem Merkmal der Staatsangehörigkeit per Zufall aus den Einwohnermelderegistern gezogen. Studienpersonen konnten sequentiell online, schriftlich sowie in den größeren Städten auch persönlich oder telefonisch mit teilweise mehrsprachigen Interviewenden an der Befragung teilnehmen. Neben Deutsch wurden Arabisch, Italienisch, Kroatisch, Polnisch und Türkisch als Studiensprachen angeboten [31].

Die Studie verfolgte das Ziel, umfassende Daten zu Gesundheitszustand, Gesundheitsverhalten, Lebensbedingungen und Inanspruchnahme von Gesundheitsleistungen zu erheben und differenzierte Aussagen nach soziodemografischen und migrationsbezogenen Merkmalen zu ermöglichen. Zudem wurden Daten zum SARS-CoV-2-Infektions- und COVID-19-Impfstatus erhoben und Fragen gestellt, die auf die indirekten sozioökonomischen Folgen der Pandemie abzielten [31].

Insgesamt nahmen 6038 Personen (2983 Frauen, 3055 Männer) an GEDA Fokus teil. Die Rücklaufquote betrug nach den Standards der *American Association for Public Opinion Research* (AAPOR) 18,4 % (Response Rate 1; [32]). Weitere Details zum Studiendesign von GEDA Fokus sind an anderer Stelle beschrieben [31].

### Indikatoren

#### Outcomevariable

Die Lebenszufriedenheit wurde anhand der Frage erfasst: „Wie zufrieden sind Sie, alles in allem, mit Ihrem Leben?“ Die Befragten wurden gebeten, sich auf einer 11-stufigen Skala (0 = „ganz und gar unzufrieden“; 10 = „ganz und gar zufrieden“) einzuordnen. Die Angaben zur Lebenszufriedenheit wurden anhand des Medians dichotomisiert. Als Outcome wurden Angaben vom Median aufwärts (7–10; kodiert mit 1) denjenigen unterhalb des Medians (0–6; kodiert mit 0), gegenübergestellt. Berichtet wird die hohe Lebenszufriedenheit (Skalenpunkte 7–10).

#### Prädiktorvariablen

##### Demografische Determinanten: Alter und Geschlecht.

Für die Variable Geschlecht wurde das gemäß Selbstangabe bei der Geburt in die Geburtsurkunde eingetragene Geschlecht herangezogen. Das Alter der Befragten wurde in die Kategorien 18 bis 39 Jahre, 40 bis 59 Jahre und 60 bis 79 Jahre eingeteilt.

##### Sozioökonomische Determinanten: Bildungsniveau und Nettoäquivalenzeinkommen.

Das Bildungsniveau wurde anhand der Selbstangaben zu schulischen und beruflichen Abschlüssen anhand der Internationalen Standardklassifikation für das Bildungswesen (*International Standard Classification of Education*, ISCED 2011 [33]) gruppiert in untere (ISCED 1–2), mittlere (ISCED 3–4) und obere (ISCED 5–8) Bildungsgruppen.

Auf die Frage: „Wie hoch ist das monatliche Netto-Einkommen Ihres Haushalts insgesamt?“, konnten Befragte entweder einen konkreten Betrag angeben oder sich in verschiedene Kategorien einordnen. Fehlende Einkommensangaben wurden mittels regressionsanalytischer Verfahren mit Informationen zu Alter, Geschlecht, Haushaltszusammensetzung, Bildung, Erwerbsstatus und beruflicher Stellung sowie mit regionalen Informationen zur Arbeitslosigkeit und Einkommenssteuer imputiert [34]. Nachfolgend wurden für die Auswertungen niedrige (Quintil 1), mittlere (Quintil 2 bis 4) und hohe Einkommensgruppen (Quintil 5) gebildet.

##### Migrationsbezogene Determinante: selbst eingeschätzte Deutschkenntnisse.

Auf die Frage: „Welche Sprache ist Ihre Muttersprache?“, konnten Befragte Deutsch oder eine andere Sprache (oder beides) angeben. Diejenigen, die Deutsch nicht als Muttersprache nannten, wurden anschließend gebeten, ihre Deutschkenntnisse auf einer 5‑stufigen Skala einzuschätzen. Für die vorliegenden Analysen wurden die Kategorien „Muttersprache/sehr gut“, „gut/mittelmäßig“ und „schlecht/sehr schlecht“ jeweils zusammengefasst.

##### Operationalisierung der indirekten sozioökonomischen Folgen der Pandemie.

Als Variable der indirekten sozioökonomischen Pandemiefolgen wurde zunächst die Veränderung in der Einkommenssituation betrachtet. Teilnehmende wurden im Anschluss an die Erfassung ihres monatlichen Haushaltsnettoeinkommens gefragt: „Hat sich das monatliche Einkommen Ihres Haushalts seit der Corona-Pandemie …“ „… verbessert“/„… verschlechtert“/„… ist es gleich geblieben?“ (3 Antwortoptionen).

Zudem wurde in GEDA Fokus ein Instrument aus der Studie „Sozio-ökonomische Faktoren und Folgen der Verbreitung des Coronavirus in Deutschland“ (SOEP-CoV-Studie; [35]) eingesetzt, um pandemiebedingte sozioökonomische Veränderungen zu erfassen. Die Teilnehmenden wurden gefragt: „Für wie groß halten Sie die Wahrscheinlichkeit, dass Sie in Folge der Maßnahmen zur Bekämpfung und langsameren Verbreitung des Corona-Virus innerhalb der nächsten 12 Monate …“ i) „… Ihren Arbeitsplatz aufgrund einer Kündigung oder Betriebsschließung verlieren werden?“, ii) „… in Zahlungsschwierigkeiten geraten werden, so dass Sie auf Sparrücklagen oder Kredite zurückgreifen müssen?“, iii) „… in ernsthafte Geldprobleme geraten werden und möglicherweise Sozialleistungen beantragen müssen?“ und iv) „… Ihren Lebensstandard drastisch einschränken müssen?“ Befragte konnten jeweils einen Prozentwert zwischen 0 % und 100 % nennen oder angeben: „Das ist bereits passiert.“ Die Antworten wurden wie folgt kategorisiert: „0 %“ (für: gänzlich unwahrscheinlich), „1–49 %“ (für: eher unwahrscheinlich), „50–100 %“ (für: eher wahrscheinlich) und: „Das ist bereits passiert.“.

### Statistische Analyse

Für alle vorgestellten Prädiktorvariablen wurden in der deskriptiven Analyse die jeweiligen Prävalenzen sowie die dazugehörigen 95 %-Konfidenzintervalle (95 %-KI) der selbst eingeschätzten hohen Lebenszufriedenheit ausgewiesen. Von einem signifikanten Unterschied zwischen den Gruppen wird ausgegangen, wenn der mittels Chi-Quadrat-Test ermittelte *p*-Wert kleiner als 0,05 ist.

Zudem wurden für alle drei Einkommensgruppen die indirekten sozioökonomischen Pandemiefolgen mittels bivariater Analysen untersucht. Dargestellt werden die Ergebnisse als Mengenflüsse (Sankey-Diagramme), die unter Verwendung des Onlinetools SankeyMATIC erstellt wurden. Abgetragen werden die gewichteten Anteile der gruppierten Wahrscheinlichkeitsangaben jeweils für alle 3 Einkommensgruppen.

Für multivariable Analysen wurden Poisson-Regressionsmodelle berechnet, um relevante Assoziationen zwischen einer hohen Lebenszufriedenheit und den vorgestellten Prädiktorvariablen zu identifizieren. Es wurden die sozioökonomischen, die migrationsbezogenen sowie die Variablen zu den indirekten sozioökonomischen Pandemiefolgen aufgrund der bivariat bestehenden Assoziationen mit der Lebenszufriedenheit eingeschlossen; für die demografischen Variablen sowie die Staatsangehörigkeit nach Einwohnermeldeamt^2^ wurde statistisch kontrolliert. Die Items des vorgestellten Instruments aus der SOEP-CoV-Studie wurden jeweils in ein separates Regressionsmodell eingeschlossen; Modell 1 beinhaltet demnach die Wahrscheinlichkeit, den Arbeitsplatz zu verlieren, Modell 2 diejenige, in Zahlungsschwierigkeiten zu geraten, Modell 3 die Wahrscheinlichkeit, Sozialleistungen beantragen zu müssen, und Modell 4 diejenige, den Lebensstandard drastisch einschränken zu müssen. Die mittels Poisson-Regression ermittelten *Prevalence Ratios* (PR) sowie die dazugehörigen 95 %-KI werden im Ergebnisteil als Forest-Plots dargestellt. Von statistisch signifikanten Assoziationen mit dem Outcome ist dann auszugehen, wenn die jeweiligen 95 %-KI den Wert 1 nicht einschließen.

Fälle mit mindestens einem fehlenden Wert in einer der untersuchten Variablen wurden aus den Analysen ausgeschlossen (*n* = 1924)^3^, was in einem finalen Analysesample von *n* = 4114 resultiert. In die Auswertungen wurde ein Gewichtungsfaktor einbezogen, der die Stichprobe hinsichtlich folgender Merkmale an die Bevölkerung mit entsprechenden Staatsangehörigkeiten angleicht: Region, Geschlecht, Alter, Bildung und Aufenthaltsdauer. Diese Randverteilungen wurden dem Mikrozensus 2018 [36] entnommen, nachdem die Daten auf die ausgewählten 5 Staatsangehörigkeitsgruppen (einschließlich doppelter Staatsangehörigkeit) eingegrenzt wurden. Um die Clusterung der Teilnehmenden innerhalb der Studienorte und die Gewichtung angemessen bei der Berechnung von Konfidenzintervallen und *p*-Werten zu berücksichtigen, wurden in allen Analysen Survey-Prozeduren für komplexe Stichproben verwendet [37].

Alle Analysen wurden mit Stata 17.0 (Stata Corp., College Station, TX, USA, 2015) durchgeführt.

## Ergebnisse

### Stichprobenbeschreibung

Die Studienpopulation ist in Tab. 1 dargestellt. Von den eingeschlossenen 4114 Teilnehmenden waren 43,5 % weiblich, der Altersmedian lag bei 38 Jahren (Range: 18–79 Jahre). 40,2 % konnten der unteren und 43,5 % der mittleren Bildungsgruppe zugeordnet werden. Der Median des monatlichen Nettoäquivalenzeinkommens lag bei 1333 €. Etwa die Hälfte berichtete muttersprachliche bzw. sehr gute Deutschkenntnisse (49,5 %). Die Mehrheit der Teilnehmenden berichtete keine Veränderungen des Haushaltsnettoeinkommens seit der Corona-Pandemie (66,7 %). Jeweils mehr als die Hälfte schätzte die Wahrscheinlichkeit für das Auftreten der vier verschiedenen indirekten sozioökonomischen Pandemiefolgen für sich selbst als gänzlich unwahrscheinlich ein (Tab. 1).

**Table 1:** 

	Studienpopulation			Anteil mit Angabe einer hohen Lebenszufriedenheit^¤^				
	% (gewichtet)	(95 %-KI)		*n* (ungewichtet)	% (gewichtet)	(95 %-KI)		*p*-Wert
*Geschlecht*								
Weiblich	43,5	40,5	46,5	1314/1930	66,4	62,4	70,2	0,122
Männlich	56,5	53,5	59,5	1434/2184	62,8	60,0	65,5	
*Alter*								
18–39 Jahre	52,8	49,9	55,7	1556/2324	65,6	62,3	68,7	0,082
40–59 Jahre	37,0	34,6	39,5	961/1415	65,1	60,6	69,4	
60–79 Jahre	10,2	8,5	12,2	231/375	55,5	46,8	63,9	
*Bildung*^*a*^								
Niedrig	40,2	36,9	43,6	617/1028	61,2	56,9	65,3	**0,018**
Mittel	43,5	40,8	46,2	1035/1567	65,5	62,5	68,4	
Hoch	16,3	14,3	18,5	1096/1519	69,2	64,5	73,6	
*Äquivalenzeinkommen*								
Niedrig	17,7	15,4	20,2	357/644	54,3	48,2	60,3	**<** **0,001**
Mittel	59,4	56,5	62,2	1484/2331	62,3	58,8	65,6	
Hoch	23,0	20,8	25,3	907/1139	77,6	73,1	81,5	
*Deutschkenntnisse*								
Muttersprache/sehr gut	49,5	45,5	53,4	1375/1892	69,3	65,6	72,8	**<** **0,001**
Gut/mittelmäßig	43,8	40,2	47,3	1181/1876	60,7	57,1	64,1	
Schlecht/sehr schlecht	6,8	5,4	8,5	192/346	52,5	42,2	62,6	
*Einkommensveränderung seit der Corona-Pandemie*								
Verbessert	8,3	6,9	10,0	341/446	71,5	63,1	78,7	**<** **0,001**
Gleich geblieben	66,7	63,9	27,9	1860/7276	67,1	64,1	69,9	
Verschlechtert	25,0	22,3	69,3	547/992	54,8	49,7	59,8	
*Wahrscheinlichkeit: Verlust des Arbeitsplatzes*								
0 %	60,7	56,6	64,7	1833/2553	69,5	66,4	72,4	**<** **0,001**
1–49 %	19,8	17,0	22,9	509/765	68,0	62,1	73,3	
50–100 %	11,9	10,1	13,9	256/480	48,3	40,7	56,0	
„Das ist bereits passiert“	7,6	5,9	9,9	150/316	39,7	31,0	49,1	
*Wahrscheinlichkeit: Zahlungsschwierigkeiten*								
0 %	54,7	50,5	58,8	1672/2272	71,8	68,9	74,6	**<** **0,001**
1–49 %	22,0	19,6	24,7	626/918	66,3	60,6	71,6	
50–100 %	14,7	12,7	16,9	291/577	47,7	40,5	55,0	
„Das ist bereits passiert“	8,6	6,8	10,8	159/347	40,7	31,4	50,6	
*Wahrscheinlichkeit: Sozialleistungen beantragen*								
0 %	55,5	51,2	59,7	1786/2392	72,3	69,5	75,0	**<** **0,001**
1–49 %	21,6	19,0	24,6	554/839	65,6	60,0	70,8	
50–100 %	13,9	11,9	16,1	248/538	44,9	38,3	51,8	
„Das ist bereits passiert“	9,0	6,9	11,7	160/345	42,3	33,2	51,9	
*Wahrscheinlichkeit: Lebensstandard einschränken*								
0 %	52,1	48,1	56,1	16.312.206	71,3	68,0	74,5	**<** **0,001**
1–49 %	21,3	18,8	24,0	630/932	66,4	61,0	71,5	
50–100 %	14,7	12,8	16,8	299/570	55,3	48,5	61,9	
„Das ist bereits passiert“	12,0	9,9	14,4	188/415	51,7	34,7	49,0	

^¤^Skalenwerte 7 bis 10

*p*-Werte aus Chi-Quadrat-Tests; **Fettdruck** statistisch signifikant

*95* *%-KI* 95 %-Konfidenzintervall

^a^Gemäß Internationaler Standardklassifikation für das Bildungswesen 2011 (ISCED 2011)

### Deskriptive Analysen

#### Lebenszufriedenheit.

Fast zwei Drittel der Teilnehmenden berichteten eine hohe Lebenszufriedenheit (64,4 %; 95 %-KI: 61,9–66,8 %). Statistisch signifikante Unterschiede nach Geschlecht oder Altersgruppen zeigten sich dabei nicht (Tab. 1). Für alle anderen Prädiktorvariablen konnten hingegen signifikante Unterschiede zwischen den Gruppen festgestellt werden, wobei insbesondere das Einkommen, die selbst eingeschätzten Deutschkenntnisse sowie die Indikatoren der indirekten sozioökonomischen Pandemiefolgen starke Zusammenhänge mit der selbst eingeschätzten Lebenszufriedenheit zeigten.

#### Indirekte sozioökonomische Pandemiefolgen für verschiedene Einkommensgruppen.

**Figure 1:**
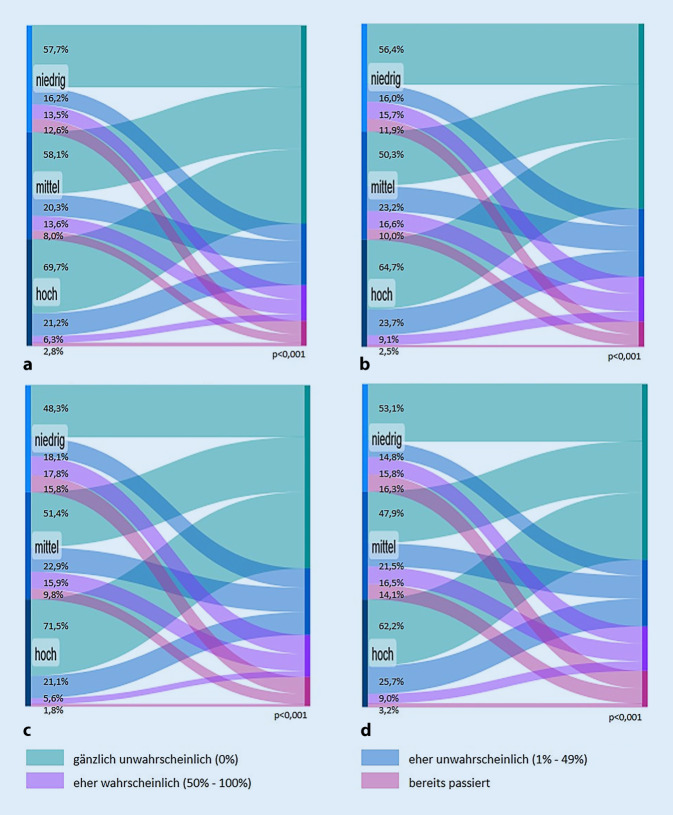

Hinsichtlich der indirekten sozioökonomischen Pandemiefolgen werden für alle vier betrachteten Items aus der SOEP-CoV-Studie Unterschiede zwischen den Einkommensgruppen deutlich (Abb. 1; Tabelle A1 im Onlinematerial). In ernsthafte Geldprobleme zu geraten und möglicherweise Sozialleistungen beantragen oder den Lebensstandard drastisch einschränken zu müssen, halten Befragte aus der hohen Einkommensgruppe häufiger für gänzlich unwahrscheinlich. Demgegenüber berichten Befragte aus der niedrigen und mittleren Einkommensgruppe bei allen betrachteten indirekten sozioökonomischen Pandemiefolgen häufiger, dass sie dies für eher wahrscheinlich halten oder dass dies bereits passiert sei. Ähnliche Zusammenhänge, wenn auch nicht für alle vier betrachteten Items der indirekten sozioökonomischen Pandemiefolgen, zeigen sich auch in Bezug auf Bildung und selbst eingeschätzte Deutschkenntnisse (Ergebnisse nicht gezeigt).

### Multivariable Poisson-Regressionen

**Figure 2:**
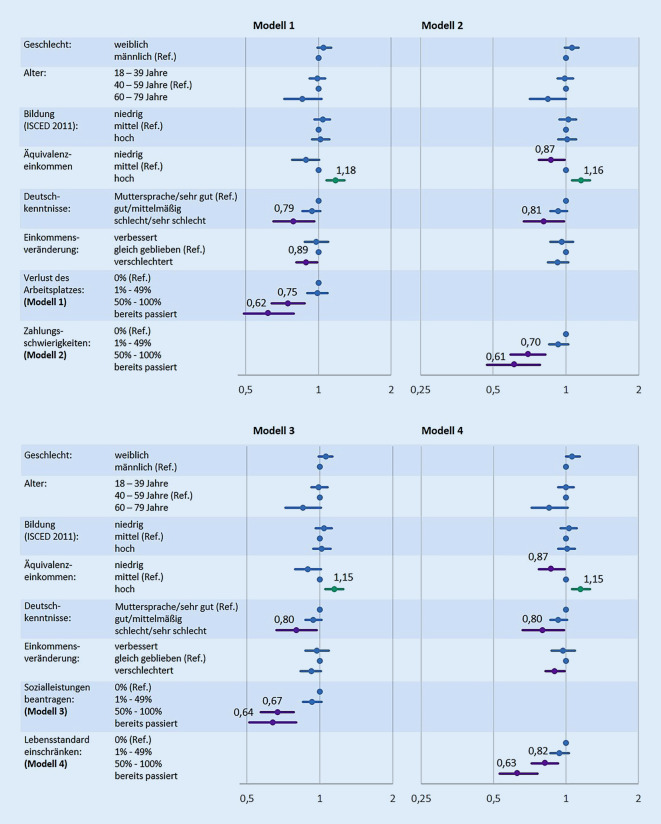

Alle vier Poisson-Regressionsmodelle zeigen einen positiven Zusammenhang zwischen einem hohen Einkommen und einer hohen Lebenszufriedenheit (Abb. 2; Tabelle A2 im Onlinematerial). Demgegenüber sind als (sehr) schlecht eingeschätzte Deutschkenntnisse jeweils negativ mit einer hohen Lebenszufriedenheit assoziiert. Auch die Einschätzung, dass die indirekten sozioökonomischen Pandemiefolgen eher wahrscheinlich eintreten werden oder bereits eingetreten sind, ist jeweils negativ mit der Lebenszufriedenheit assoziiert. Negative Zusammenhänge mit der Lebenszufriedenheit zeigen sich ebenfalls für ein niedriges Einkommen sowie mit der Verschlechterung des Einkommens im Zuge der Pandemie, wenn auch nicht in allen vier Modellen.

## Diskussion

Die vorliegenden Analysen konnten in einer Stichprobe von Menschen mit ausgewählten Staatsangehörigkeiten Zusammenhänge zwischen der Lebenszufriedenheit und dem Nettoäquivalenzeinkommen, den selbst eingeschätzten Deutschkenntnissen sowie erstmals auch den indirekten sozioökonomischen Pandemiefolgen zeigen. Insgesamt zeigte sich, dass die Mehrheit der Befragten eine hohe Lebenszufriedenheit berichtet. Im Vergleich zur Studie „Corona-Monitoring bundesweit – Welle 2“ (November 2021–Februar 2022) war sie jedoch geringer. Hier gaben 81,3 % der 18- bis 79-Jährigen einen Wert von über 6 an; auch der Median der selbst eingeschätzten Lebenszufriedenheit lag in dieser Studie mit 8 um einen Punkt höher als im vorliegenden Sample (eigene Berechnungen).

Es wurde deutlich, dass Befragte aus der mittleren und niedrigen Einkommensgruppe die indirekten Folgen der Pandemie häufiger für wahrscheinlicher hielten (bzw. diese sich bereits in ihrem Leben manifestiert hatten) als die Befragten aus der hohen Einkommensgruppe. Ein sozialer Gradient, der bereits eingangs für die Allgemeinbevölkerung beschrieben wurde [16, 17], hat sich auch in einer Stichprobe von Menschen mit ausgewählten Staatsangehörigkeiten bestätigt.

Zusammenhänge zwischen der Einkommenssituation und der Lebenszufriedenheit sind in der Literatur beschrieben [26, 38, 39], wobei ein höheres Einkommen mit einer höheren Lebenszufriedenheit einhergeht. Ein hohes Einkommen dürfte dadurch, dass Grundbedürfnisse gedeckt sind, zu einem weniger sorgenvollen Leben beitragen und damit positiv auf die Lebenszufriedenheit wirken [39, 40]. Demgegenüber sind, wie erwähnt, Zusammenhänge zwischen Armut und schlechteren Gesundheitsoutcomes vielfach belegt [25].

Dass bessere Deutschkenntnisse mit einer höheren Lebenszufriedenheit einhergehen, zeigte sich sowohl in einer Studie unter Arbeitsmigrant:innen in Österreich [41] als auch unter Geflüchteten in Deutschland [42], jedoch zu präpandemischen Zeiten. Deutschkenntnisse stellen einen Indikator für soziale Teilhabe dar; sie ermöglichen besseren Zugang zu Informationen, zu Rechten und auch zur Gesundheitsversorgung [6]. Geringere Deutschkenntnisse können demnach auch mit struktureller Benachteiligung und systematischen Ausschlüssen einhergehen, welche sich wiederum auf die Lebenszufriedenheit auswirken können. Gerade in Krisenzeiten kann dies Unsicherheiten verstärken, auch dadurch, dass Menschen sich die zumeist auf Deutsch angebotenen Informationen in Bezug auf die Pandemie (z. B. Informationen zu COVID-19 allgemein, aber auch zu staatlichen Unterstützungsangeboten) vermutlich weniger gut zunutze machen können.

Von Einkommens- und Arbeitsplatzverlusten im Zuge der Pandemie sind einem Bericht der Organisation für wirtschaftliche Zusammenarbeit und Entwicklung (OECD) zufolge vor allem ohnehin benachteiligte Bevölkerungsgruppen betroffen, wie Jüngere, gering Qualifizierte oder auch Migrant:innen [43]. Ergebnisse des Deutschen Instituts für Wirtschaftsforschung (DIW) verweisen auch in Bezug auf die Allgemeinbevölkerung in Deutschland darauf, dass insbesondere Menschen mit geringerer Bildung oder niedrigem Einkommen von indirekten sozioökonomischen Folgen der Pandemie betroffen waren [44]. Wie eingangs erwähnt, bestätigen dies Daten aus Deutschland auch für Menschen mit *Migrationshintergrund* [19, 22, 23]. Hinsichtlich der Betroffenheit von den indirekten sozioökonomischen Pandemiefolgen nach Einkommensgruppen bestätigen die vorliegenden Analysen diese Befunde auch erstmals innerhalb einer Stichprobe von Menschen mit ausgewählten Staatsangehörigkeiten. Gleichzeitig sehen wir in den vorliegenden Daten jedoch ohnehin eine Einkommensbenachteiligung. Während der Median des Nettoäquivalenzeinkommens der 18- bis 79-Jährigen deutschsprachigen Allgemeinbevölkerung in der Studie „GEDA 2019/2020“ 1700 € betrug (eigene Berechnungen), lag er in der vorliegenden Stichprobe mit 1333 € mehr als 20 % niedriger.

Dass Einkommenseinbußen oder Arbeitsplatzverluste einen Einfluss auf die Lebenszufriedenheit haben, zeigen sowohl Ergebnisse aus Deutschland [45–47] als auch internationale Befunde [48–50]. Langfristig kann sich eine geringere Lebenszufriedenheit auf das psychische Wohlbefinden [26–30], z. B. in Form von Depressivität oder chronischem Stress, und damit auch auf die körperliche Gesundheit [27, 29] niederschlagen. Die vorliegenden Ergebnisse geben Hinweise darauf, dass Verhältnisprävention vermehrt in den Fokus rücken sollte. Planbarkeit der sozioökonomischen Lebensbedingungen, wie Arbeitsplatz- oder Einkommenssicherheit, sollte für alle Menschen gleichermaßen gewährleistet sein, um gesundheitlichen Ungleichheiten entgegenzuwirken. Gleichzeitig sollten Aspekte wie strukturelle und alltägliche Diskriminierung adressiert werden, die ebenfalls auf die Gesundheit wirken [6], zusätzlich aber auch mit Vertrauensverlusten in öffentliche Institutionen und behördliche Maßnahmen einhergehen und so als Katalysator für Zukunftsängste und verminderte Lebenszufriedenheit wirken können, die indirekt mit einer Reihe zentraler Gesundheitsoutcomes im Zusammenhang stehen. Um dem entgegenzuwirken, sollten Unterstützungs- und Beratungsangebote mehrsprachig, communityorientiert und aufsuchend gestaltet werden, um gerade in Zeiten der Unsicherheit ohnehin bestehende Ungleichheiten nicht noch weiter zu verschärfen.

Limitierend ist hinsichtlich der vorliegenden Ergebnisse anzumerken, dass anhand von Querschnittsdaten keinerlei Rückschlüsse auf die Wirkrichtung von Zusammenhängen möglich sind. Entsprechend wäre es wichtig, die beobachteten Zusammenhänge auch langfristig in Kohortenstudien zu untersuchen. Hinsichtlich der Wahrscheinlichkeit des Arbeitsplatzverlustes ist anzumerken, dass nicht für alle Befragten bekannt ist, ob sie zum Befragungszeitpunkt tatsächlich erwerbstätig waren, da nach der Haupttätigkeit gefragt wurde. Gleichwohl können auch Menschen in Rente oder auch Studierende nebenher erwerbstätig sein, weshalb auf eine Einschränkung der Stichprobe auf Haupterwerbstätige verzichtet wurde. Eine weitere Limitation besteht darin, dass wir anhand der vorliegenden Daten lediglich Aussagen über Menschen mit den ausgewählten Staatsangehörigkeiten, nicht jedoch über alle Menschen mit Migrationsgeschichte in Deutschland treffen können. Dennoch handelt es sich hierbei um große Gruppen unter den hierzulande lebenden Menschen mit Migrationsgeschichte und das bisher größte migrationsspezifische Sample in Deutschland, welches tiefgehende Analysen zwischen der gesundheitlichen Lage, migrationsbezogenen, sozialen und strukturellen Determinanten der Gesundheit ermöglicht. Gleichzeitig konnten wir anhand dieses Samples erstmals für den deutschen Kontext zeigen, dass sich auch bei Menschen mit Migrationsgeschichte Ungleichheiten hinsichtlich der indirekten sozioökonomischen Pandemiefolgen und deren Auswirkungen auf die Lebenszufriedenheit zeigen.

## Fazit

Der vorliegende Beitrag konnte die Ungleichverteilung indirekter Pandemiefolgen, wie Arbeitsplatz- oder Einkommensverluste, die für die Allgemeinbevölkerung bereits bekannt ist, auch in einer Stichprobe von Menschen mit ausgewählten Staatsangehörigkeiten zeigen. Darüber hinaus konnte der Beitrag nachweisen, dass die Lebenszufriedenheit – ein Faktor, der für eine Reihe von gesundheitlichen Outcomes relevant ist – bei Personen geringer ist, die von diesen Auswirkungen betroffen sind oder diese mit einer hohen Wahrscheinlichkeit antizipieren, als bei denjenigen, die keine Arbeitsplatz- oder Einkommensverluste befürchten. Da eine geringere Lebenszufriedenheit auch mit Folgen für die allgemeine und psychische Gesundheit einhergeht, gilt es, strukturelle Ursachen sozioökonomischer und sozialer Benachteiligungen abzubauen, um damit auch gesundheitliche Ungleichheiten nachhaltig zu adressieren und für künftige Krisen als gesamte Gesellschaft besser vorbereitet zu sein.

## Supplementary Information





## Footnotes

1 Im Beitrag wird der Begriff „Menschen mit Migrationsgeschichte“ verwendet und meint damit alle Menschen, die einen eigenen oder familiären biografischen Bezug zu Migration oder Flucht haben. Der mittlerweile in der Kritik stehende und häufig als diskriminierend empfundene Begriff „Menschen mit Migrationshintergrund*“* wird hingegen verwendet, wenn damit statistisch definierte Personengruppen beschrieben werden. Weitere Ausführungen hierzu finden sich in [6].

2 Auf eine Darstellung der Ergebnisse zur statistischen Kontrolle der demografischen Variablen und der Staatsangehörigkeit wird mangels Assoziationen verzichtet. Zudem verzichten wir auf die Ausweisung von Ergebnissen nach Staatsangehörigkeitsgruppen, da sich zum einen die Stichprobenzusammensetzung vermutlich systematisch zwischen den einzelnen Gruppen unterscheidet und ihre Vergleichbarkeit daher erschwert ist (z. B. haben Menschen mit italienischer Staatsangehörigkeit häufiger selbstinitiativ direkt zu Studienbeginn teilgenommen, durch die Hausbesuche wurden jedoch im späteren Studienverlauf häufiger Menschen mit niedrigerer Bildung und einer schlechteren subjektiven Gesundheit erreicht). Zum anderen birgt der Vergleich die Gefahr, bei Beschreibung einzelner Effekte je nach Staatsangehörigkeit pauschalisierend und stereotypisierend zu sein.

3 Separate Missing-Kategorien wurden aufgrund möglicherweise systematisch verzerrten Antwortverhaltens nicht mitgeführt, zudem sind diese Kategorien unzureichend interpretierbar.
