# Supplementary material for: People with a history of migration in the COVID-19 pandemic: Associations between life satisfaction and indirect socioeconomic effects of the pandemic
Source: Bundesgesundheitsblatt Gesundheitsforschung Gesundheitsschutz. 2023 Jul 20;66(8):901–10. [Article in German] doi: 10.1007/s00103-023-03741-0 (PMC10371942; doi:10.1007/s00103-023-03741-0)
Supplement: Supplementary file 1 [file 103_2023_3741_MOESM1_ESM.pdf]

Onlinematerial

Tabelle A1: Zusammenhänge zwischen der Höhe des Äquivalenzeinkommens und der eingeschätzten Wahrscheinlichkeit, selbst von den vier verschiedenen indirekten sozioökonomischen Folgen der Pandemie (a-d) betroffen zu sein – Ergebnisse bivariater Analysen; GEDA Fokus (2021-2022), n=4.114.

	Äquivalenzeinkommen								
	niedrig			mittel			hoch		
	% (gewichtet)	95%-KI		% (gewichtet)	95%-KI		% (gewichtet)	95%-KI	
Verlust des Arbeitsplatzes									
0%	57,7%	49,2	65,8	58,1%	53,2	62,9	69,7%	64,7	74,3
1% - 49%	16,2%	11,1	23,0	20,3%	16,8	24,2	21,2%	17,1	25,9
50 - 100%	13,5%	9,9	18,3	13,6%	11,3	16,3	6,3%	4,3	9,0
„Das ist bereits passiert“	12,6%	8,6	17,9	8,0%	5,7	11,1	2,8%	1,5	5,2
Zahlungsschwierigkeiten									
0%	56,4%	48,5	64,1	50,3%	45,2	55,3	64,7%	59,5	69,7
1% - 49%	16,0%	11,8	21,3	23,2%	19,7	27,2	23,7%	19,6	28,2
50 - 100%	15,7%	11,4	21,2	16,6%	13,9	19,6	9,1%	6,5	12,6
„Das ist bereits passiert“	11,9%	8,0	17,3	10,0%	7,7	12,9	2,5%	1,4	4,6
Sozialleistungen beantragen									
0%	48,3%	39,9	56,9	51,4%	46,0	56,8	71,5%	66,4	76,1
1% - 49%	18,1%	13,1	24,6	22,9%	19,3	27,0	21,1%	17,1	25,7
50 - 100%	17,6%	13,8	22,5	15,9%	13,3	19,0	5,6%	3,6	8,6
„Das ist bereits passiert“	15,8%	11,3	21,6	9,8%	6,8	13,9	1,8%	0,9	3,6
Lebensstandard einschränken									
0%	53,1%	44,2	61,8	47,9%	42,9	52,9	62,2%	56,2	67,9
1% - 49%	14,8%	10,4	20,7	21,5%	18,4	24,9	25,7%	20,8	31,2
50 - 100%	15,8%	11,6	21,2	16,5%	14,2	19,2	9,0%	6,3	12,8
„Das ist bereits passiert“	16,3%	12,0	21,9	14,1%	11,3	17,6	3,2%	1,8	5,4

95%-KI: 95%-Konfidenzintervalle

Tabelle A2: Mit der Angabe einer hohen Lebenszufriedenheit assoziierte Faktoren - Ergebnisse der multivariablen Poisson-Regressionen; GEDA Fokus (2021-2022), n=4.114

	**Modell 1**				**Modell 2**				**Modell 3**				**Modell 4**			
	PR	(95%-KI)		p-Wert	PR	(95%-KI)		p-Wert	PR	(95%-KI)		p-Wert	PR	(95%-KI)		p-Wert
Geschlecht																
weiblich	1,05	0,99	1,13	0,125	1,06	0,99	1,13	0.088	1,06	0,99	1,13	0,105	1,06	1,00	1,14	0,063
männlich	Ref.				Ref.				Ref.				Ref.			
Alter																
18 - 39 Jahre	0,99	0,92	1,07	0,850	0,99	0,92	1,07	0,858	0,99	0,92	1,08	0,871	1,00	0,93	1,08	0,959
40 - 59 Jahre	Ref.				Ref.				Ref.				Ref.			
60 - 79 Jahre	0,86	0,72	1,03	0,096	0,84	0,71	1,00	0,046	0,85	0,72	1,01	0,063	0,85	0,72	1,02	0,073
Bildung (ISCED 2011)																
niedrig	1,04	0,96	1,12	0,018	1,02	0,94	1,10	0,632	1,04	0,96	1,12	0,327	1,03	0,95	1,11	0,508
mittel	Ref.				Ref.				Ref.				Ref.			
hoch	1,02	0,94	1,11	0,561	1,01	0,93	1,10	0,727	1,02	0,94	1,11	0,674	1,01	0,93	1,09	0,852
Äquivalenzeinkommen																
niedrig	0,89	0,78	1,01	0,061	**0,87**	**0,77**	**0,99**	**0,032**	0,89	0,79	1,01	0,062	**0,87**	**0,77**	**0,99**	**0,034**
mittel	Ref.				Ref.				Ref.				Ref.			
hoch	**1,18**	**1,08**	**1,28**	**<0,001**	**1,16**	**1,06**	**1,26**	**0,001**	**1,15**	**1,05**	**1,25**	**0,002**	**1,15**	**1,06**	**1,26**	**0,001**
Deutschkenntnisse																
Muttersprache/ sehr gut	Ref.				Ref.				Ref.				Ref.			
gut/ mittelmäßig	0,94	0,86	1,02	0,111	0,93	0,86	1,01	0,098	0,94	0,87	1,02	0,121	0,93	0,86	1,01	0,087
schlecht/ sehr schlecht	**0,79**	**0,65**	**0,96**	**0,020**	**0,81**	**0,67**	**0,98**	**0,031**	**0,80**	**0,66**	**0,97**	**0,022**	**0,80**	**0,66**	**0,98**	**0,028**
Einkommensveränderung seit der Corona-Pandemie																
verbessert	0,98	0,88	1,10	0,737	0,96	0,86	1,07	0,480	0,97	0,87	1,09	0,601	0,97	0,87	1,09	0,602
gleich geblieben	Ref.				Ref.				Ref.				Ref.			
verschlechtert	**0,89**	**0,81**	**0,99**	**0,027**	0,92	0,84	1,02	0,124	0,92	0,83	1,01	0,084	**0,90**	**0,82**	**0,99**	**0,038**
Verlust des Arbeitsplatzes																
0%	Ref.															
1% - 49%	0,99	0,90	1,09	0,839												
50 - 100%	**0,75**	**0,64**	**0,88**	**0,001**												
„Das ist bereits passiert“	**0,62**	**0,49**	**0,79**	**<0,001**												
Zahlungsschwierigkeiten																
0%					Ref.											
1% - 49%					0,93	0,85	1,02	0,114								
50 - 100%					**0,70**	**0,59**	**0,82**	**<0,001**								
„Das ist bereits passiert“					**0,61**	**0,47**	**0,78**	**<0,001**								
Sozialleistungen beantragen																
0%									Ref.							
1% - 49%									0,93	0,85	1,02	0,113				
50 - 100%									**0,67**	**0,57**	**0,78**	**<0,001**				
„Das ist bereits passiert“									**0,64**	**0,51**	**0,80**	**<0,001**				
Lebensstandard einschränken																
0%													Ref.			
1% - 49%													0,94	0,86	1,03	0,207
50 - 100%													**0,82**	**0,72**	**0,93**	**0,003**
„Das ist bereits passiert“													**0,63**	**0,53**	**0,76**	**<0,001**

Ref.: Referenzkategorie; PR: *Prevalence Ratios;* 95%-KI: 95%-Konfidenzintervalle; Fettdruck: statistisch signifikant; alle Modelle adjustiert für Staatsangehörigkeit nach Einwohnermelderegister.
